# Renal Autotransplantation After Yang–Monti Neoureter Procedure: Surgical Case Report and Brief Literature Review

**DOI:** 10.1155/criu/1351432

**Published:** 2025-12-16

**Authors:** Reece M. Anderson, Alex Chang, Wesley Baas

**Affiliations:** ^1^ Department of Urology, University of Cincinnati Medical Center, Cincinnati, Ohio, USA, uc.edu; ^2^ Division of Transplant Surgery, Department of Surgery, University of Cincinnati Medical Center, Cincinnati, Ohio, USA, uc.edu

**Keywords:** autografts, hydronephrosis, ureteral obstruction

## Abstract

**Purpose:**

Ureteral strictures are a well‐documented pathology in urology, commonly resulting from congenital anomalies, iatrogenic injury, or other acquired conditions such as infection, trauma, or radiation. While ischemic and nonischemic strictures are recognized, bilateral idiopathic ureteral strictures remain rare, particularly when associated with systemic thrombotic microangiopathy. This case report describes a 25‐year‐old female with a history of IgA deficiency and atypical hemolytic uremic syndrome (aHUS), who developed bilateral ureteral strictures following envenomation by a brown recluse spider. The purpose of this report is to highlight an unusual cause of ureteral stricture formation, discuss its underlying pathophysiology, and evaluate long‐term surgical outcomes, including the failure of a Yang–Monti neoureter and the subsequent necessity for left renal autotransplantation.

**Results:**

The patient initially developed bilateral ureteral ischemia and subsequent stricture formation, requiring percutaneous nephrostomy tubes for urinary drainage. The left ureter exhibited an 8–9 cm occlusion, leading to the decision to perform a Yang–Monti neoureter using a jejunal interposition graft. Despite initial surgical success, the patient later presented with left‐sided hydronephrosis and recurrent urinary tract infections, revealing complete stricture of the neoureter. After thorough evaluation, she underwent left renal autotransplantation with ureteral reimplantation. Postoperative outcomes were favorable, with stable renal function, although complicated by transient ileus. This case underscores the challenges of managing extensive ureteral stricture disease, particularly when secondary to systemic microangiopathic processes triggered by envenomation.

**Conclusions:**

To our knowledge, this is the first documented case of long‐segment ureteral stricture as a sequela of brown recluse spider envenomation. The venom’s prothrombotic and endothelial‐damaging effects likely contributed to progressive ischemic injury and fibrosis, leading to bilateral stricture formation. While the Yang–Monti technique provided temporary urinary diversion, its long‐term viability was limited, necessitating a definitive solution via renal autotransplantation. This case highlights the need for increased awareness of envenomation‐related ischemic complications, particularly in patients presenting with delayed‐onset ureteral obstruction. Future research should further explore envenomation‐induced microangiopathy and optimal surgical management strategies for complex ureteral stricture disease.

## 1. Introduction

Ureteral strictures are common pathologic findings in urology which can be congenital—such as ureteropelvic junction (UPJ) obstructions—or acquired. Acquired ureteral strictures are most commonly secondary to iatrogenic (35%) and benign etiologies (35%) including impacted calculi, retroperitoneal fibrosis, or infections [1]. Idiopathic ureteral strictures are common and account for about 20% of all ureteral strictures. However, there are few cases of bilateral, idiopathic ureteral strictures in the literature [2]. Ureteral strictures are further classified as either ischemic or nonischemic and typically occur as a result of either open surgery or radiation, although there is not a consensus regarding definition of this condition [1]. Ischemic ureteral strictures typically manifest as unilateral and most frequently occur in the midureteral segment in watershed areas of blood supply to the ureter.

There are many options for surgical management of ureteral strictures. Endoscopic management of strictures can involve more conservative methods such as indwelling ureteral stents and nephrostomy tubes for short‐term management. Endoscopic surgical techniques such as balloon dilation and endoureterotomy have demonstrated to be effective methods for short‐segment strictures but have low success rates for long‐segment (≥ 2 cm) strictures [3]. Long‐segment strictures often require more involved surgical management which can include minimally invasive techniques or open surgery. Minimally invasive techniques such as robot‐assisted buccal graft ureteroplasty are most often used for strictures ranging from 3 to 6 cm, but this method has been reported for strictures as long as 11 cm [4]. This technique has high success rates (ranging from 67% to 100%) but is highly dependent on vascular supply for short and long‐term success [4]. More invasive open techniques like Boari flaps and psoas hitch are most commonly reported in patients with distal and midureteral defects [5]. The Yang–Monti method for creation of a neoureter using ileal interposition is an uncommon technique for urinary diversion which has demonstrated some degree of efficacy in whole ureter replacement [6, 7]. This technique has been shown to be a reasonable alternative for urinary diversion in select patient populations—particularly those who do not wish to undergo recurrent stent changes, percutaneous nephrostomy. Renal autotransplantation is generally considered to be the last alternative to nephrectomy for patients with ureteral stricture disease as it represents a technically challenging surgery and carries risk of ischemic/reperfusion injury to the transplanted organ [4].

Here, we describe the case of a patient who developed bilateral ureteral strictures secondary to a systemic, complement‐mediated inflammatory process which resulted in ischemic necrosis with stricture formation in bilateral ureters who had a late failure of the Yang–Monti neoureter and ultimately required autotransplantation of the left kidney. This manuscript was prepared in accordance with the 2023 SCARE guidelines for surgical case reporting [8].

## 2. Patient Information

Here, we present a 25‐year‐old, white female with past medical history significant for IgA deficiency and hypertension. She initially presented with thrombotic microangiopathy with large vessel thrombosis secondary to atypical hemolytic uremic syndrome (aHUS) following envenomation from a brown recluse spider bite (May 2017). She presented with ischemic injury to bilateral ureters and colonic necrosis (requiring subsequent subtotal colectomy). Mechanism of injury involving complement mediated inflammatory reaction and thrombotic microangiopathy which resulted in ischemic infarction of the colon, small bowel, and bilateral ureters—long‐segment stricture of the left ureter and a focal stricture on the right, mid ureter. She was started on eculizumab to limit ongoing complement‐mediated injury. The left ureter obstruction extended from the UPJ to the distal ureter. Urinary drainage was initially achieved with percutaneous nephrostomy tubes (PCN). Cystourethroscopy with antegrade and retrograde pyelography demonstrated an 8–9 cm of occlusion at the proximal ureter with complete occlusion distal to the UPJ.

Discussion for management options of the left ureteral stricture included Yang–Monti neoureter, autotransplantation of the left kidney, long‐term PCN, and nephrectomy. At the time, our patient was an avid horseback rider and was counseled on the risks of injury to a low‐lying kidney if she were to opt for autotransplantation. She and her family ultimately decided to proceed with the Yang–Monti neoureter which was subsequently performed using a 3‐cm segment of pedicled jejunal interposition which was rotated to the left retroperitoneum and tubularized around a 14‐Fr catheter using the Yang–Monti technique.

**Figure 1:**
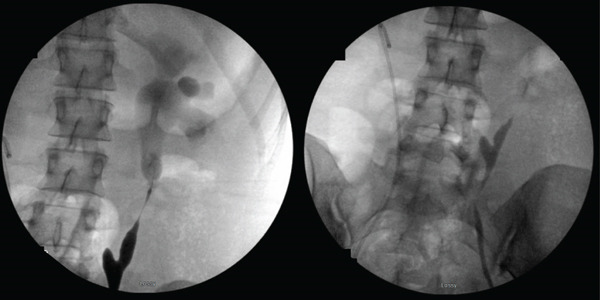
Fluoroscopy demonstrating complete stricture of the Yang–Monti conduit—June 2024.

In July 2023, the patient presented with left‐sided hydronephrosis, recurrent UTIs, and opioid‐induced constipation. At that time, MAG3 Lasix renogram demonstrated preservation of renal function bilaterally with a 53% function on the left and 47% function on the right. Approximately 2 months later, she was found to have new right‐sided hydronephrosis associated with a proximal ureteral stricture. In November 2023, she underwent robotic, right ureteroplasty with buccal mucosal graft. In June 2024, ureteroscopy revealed satisfactory drainage of her right ureter and new left‐sided hydronephrosis. Imaging studies revealed complete stricture of the neoureter bowel segment indicating failure of the Yang–Monti conduit (Figure 1). Of note, the patient underwent extensive workup for inflammatory bowel disease including upper and lower GI tract endoscopy with biopsies taken from her esophagus, gastric antrum/body, duodenum, and rectum, as well as histopathologic examination of her colon following right hemicolectomy. Microscopic examination demonstrated evidence of acute ischemic injury secondary to thrombotic angiopathy and “cannot be ascribed to IBD (Crohn disease or ulcerative colitis).”

In December 2024, the patient underwent open left nephroureterectomy, resection of jejunal interposition with autotransplantation. The previously created Yang–Monti jejunal conduit was identified. After removal of the indwelling stent, there was no visible lumen within the Yang–Monti conduit. The remaining native ureter was dilated above the sclerotic conduit. The collecting system was mildly dilated. The left kidney was removed from the patient and flushed in 4°C Belzer solution. The parenchyma appeared normal. The PCN was removed and the site was closed with absorbable suture. The autograft was implanted on the left external iliac artery and vein. After reperfusion of the kidney, brisk urine was noted from the autograft. A full‐thickness anastomosis of the UPJ to the dome of the bladder was created over a 7‐Fr ureteral stent.

Postoperative course was complicated by prolonged ileus. Creatinine nadir to 0.69 on Postoperative Day 6. Duplex of the autograft demonstrated mild pelvocaliectasis with renal artery resistive indices between 0.53 and 0.64. She was discharged to home in good condition on Postoperative Day 7. Postoperative follow‐up labs and renal ultrasound demonstrated excellent kidney function at 6 months with creatinine baseline at 0.9 mg/dL, consistent with her pre‐op baseline.

## 3. Discussion

Hemolytic uremic syndrome (HUS) has been identified as a rare but serious complication following envenomation by the brown recluse spider (*Loxosceles reclusa*) [9, 10]. The brown recluse venom contains sphingomyelinase D which is a potent enzyme known to induce tissue necrosis, vascular endothelial damage, and a prothrombotic state which may contribute to ischemic injury and fibrosis of the ureter and other visceral organs [11]. While the connection between HUS and *Loxosceles* envenomation is established, the pathway leading from envenomation to ureteral fibrosis and stricture formation remains unclear and warrants further investigation. Given that ischemia and inflammation are known contributors to stricture formation in other contexts (e.g., radiation, chronic infection, and iatrogenic injury), it is plausible that the systemic vascular effects of *Loxosceles* venom create a similar pathophysiologic process within the ureter [12]. The progression from acute envenomation to delayed ureteral fibrosis could be due to microvascular thrombosis, direct endothelial injury, or an exaggerated immune‐mediated fibrotic response, all of which have been observed in cases of cutaneous and systemic loxoscelism [13]. To our knowledge, this report is the first to document long‐segment ureteral stricture as a sequela to brown recluse spider bite.

The current body of research on ureteral stricture etiology primarily focuses on other causes, such as kidney stones, tumors, infections, and iatrogenic injuries [14]. The addition of brown recluse spider envenomation as a potential, albeit rare, cause of ureteral stricture would expand the understanding of this condition’s risk factors and inform clinical practice in the diagnosis and management of ureteral strictures, particularly in regions where the brown recluse spider is endemic. This case underscores the importance of considering envenomation‐related ischemic damage in patients presenting with delayed‐onset ureteral obstruction following a brown recluse spider bite. Future research should be aimed at characterizing the incidence, pathophysiology, and optimal management of envenomation‐induced ureteral strictures. Additionally, raising awareness among urologists, nephrologists, and emergency medicine physicians may facilitate earlier diagnosis and intervention, potentially mitigating the progression to long‐term renal dysfunction.

## 4. Review of Literature

The Yang–Monti technique was first described by Monti et al. in 1997 as a variation of the Mitrofanoff principle which allows for urinary diversion from the renal collecting system into the bladder through small bowel interposition. This option provides a long‐term solution for patients who are not suitable for frequent stent exchanges or long‐term nephrostomy tubes. This technique has been shown to be a safe and effective method for urinary diversion; however, long‐term outcomes have been mixed. A 2017 case series and systematic review evaluated a cohort of 269 patients who underwent ureteral reconstruction with ileal interposition [15]. This study found long‐term complication prevalence of fistula (4.7%) and stricture (4.1%) development. The most likely mechanism by which these conduits ultimately fail may result from excessive mobilization of the small bowel segments—in combination with retubularization—leading to chronic ischemia and ultimately promoting stricture formation.

Albeit infrequent, Yang–Monti conduit stricture should be considered as a possible outcome which may require additional intervention. Surgical options for definitive management of ureteroileal strictures can involve endoscopic techniques such as metallic or silicone ureteral stents and balloon dilation. Endoureterotomy is an option in native ureters but is considered a contraindication for management of ureteroileal strictures [1]. A 2013 review comparing endoscopic versus open techniques for ureteroileal strictures following cystectomy found overall better results for open techniques, particularly for patients with strictures greater than 1 cm [16]. Moreover, this study found better success rates for open versus endoscopic interventions for strictures < 1 cm (100% vs. 50%, respectively). Open techniques, which are inherently more invasive and morbid procedures, are typically reserved for patients with favorable preoperative characteristics including kidney function, overall morbidity, body habitus, and life expectancy. For patients with conduit failure and good preoperative renal function, nephrectomy with renal autotransplantation may be considered as a safe and effective option for definitive management of long‐segment ureteral strictures.

In summary, the Yang–Monti principle technique offers a valuable surgical option for urinary diversion in specific patient populations. However, the potential for both short‐term and long‐term complications, coupled with the risk of kidney function deterioration in patients with pre‐existing renal impairment, necessitates a thorough discussion regarding risks and benefits with the patient and thorough discussion about potential for repeat operations for definitive management. Further research and long‐term follow‐up studies are warranted to optimize patient outcomes and refine the selection criteria for this surgical intervention.

## Consent

The patient and her family provided enthusiastic and informed consent for the write‐up and publication of her case.

## Conflicts of Interest

The authors declare no conflicts of interest.

## Funding

No funding was received for this manuscript.

## Data Availability

The data that support the findings of this study are available on request from the corresponding author. The data are not publicly available due to privacy or ethical restrictions.
